# Prediction model for irreversible intestinal ischemia in strangulated bowel obstruction

**DOI:** 10.1186/s12893-022-01769-8

**Published:** 2022-08-22

**Authors:** Toshimichi Kobayashi, Naokazu Chiba, Itsuki Koganezawa, Masashi Nakagawa, Kei Yokozuka, Shigeto Ochiai, Takahiro Gunji, Toru Sano, Koichi Tomita, Satoshi Tabuchi, Eiji Hidaka, Shigeyuki Kawachi

**Affiliations:** Department of Digestive and Transplantation Surgery, Tokyo Medical University Hachioji Medical Center, Tokyo, Japan

**Keywords:** Bowel strangulation, Intestinal ischemia, Prediction, Computed tomography, Surgical emergency, Preoperative diagnosis

## Abstract

**Background:**

Preoperatively diagnosing irreversible intestinal ischemia in patients with strangulated bowel obstruction is difficult. Therefore, this study aimed to establish a prediction model for irreversible intestinal ischemia in strangulated bowel obstruction.

**Methods:**

We included 83 patients who underwent emergency surgery for strangulated bowel obstruction between January 2014 and March 2022. The predictors of irreversible intestinal ischemia in strangulated bowel obstruction were identified using logistic regression analysis, and a prediction model for irreversible intestinal ischemia in strangulated bowel obstruction was established using the regression coefficients. Receiver operating characteristic analysis and fivefold cross-validation was used to assess the model.

**Results:**

The prediction model (range, 0–4) was established using a white blood cell count of ≥ 12,000/µL and the computed tomography value of peritoneal fluid that was ≥ 20 Hounsfield units. The areas of the receiver operating characteristic curve of the new prediction model were 0.814 and 0.807 after fivefold cross-validation. A score of ≥ 2 was strongly suggestive of irreversible intestinal ischemia in strangulated bowel obstruction and necessitated bowel resection (odds ratio = 15.938). The bowel resection rates for the prediction scores of 0, 2, and 4 were 15.2%, 66.7%, and 85.0%, respectively.

**Conclusion:**

Our model may help predict irreversible intestinal ischemia that necessitates bowel resection for strangulated bowel obstruction cases and thus enable surgeons to recognize the severity of the situation, prepare for deterioration of patients with progression of intestinal ischemia, and select the appropriate surgical procedure for treatment.

## Background

Strangulated bowel obstruction (SBO) is defined as bowel obstruction with compromised intestinal blood flow and can be caused by a fibrous cord, torsion, internal hernia, or adhesions due to previous abdominal surgery [1]. SBO is a serious condition requiring early diagnosis and immediate surgery because intestinal ischemia (II) due to bowel strangulation can lead to bowel necrosis and even perforation, eventually causing septic shock. Particularly, as for SBO with irreversible II, a delayed intervention has a high risk of mortality, and the mortality rate for SBO is reportedly 16% in patients with irreversible II, compared to 3% in patients with reversible II [2]. Identifying patients with irreversible II is vital to understanding the severity and managing the deteriorating condition, including perioperative intensive care.

Computed tomography (CT) can detect SBO with a sensitivity ranging from 73 to 100% and a specificity ranging from 61 to 100% [3]. However, to distinguish between irreversible and reversible II in SBO preoperatively remains challenging. Regardless of the experience or seniority of the surgeon, physical examination for the detection of strangulation has a success rate of only 48% [4]. Moreover, despite advances in imaging, patients requiring emergency surgery are usually poor candidates for such examinations. Particularly, reduced bowel wall enhancement on contrast-enhanced CT is reportedly a significant predictive factor [5–7]. However, contrast agents are contraindicated in some patients due to severe renal dysfunction or iodine allergy. Additionally, assessing the presence or absence of bowel wall enhancement highly depends on clinicians and has a poor objective value. Therefore, this study aimed to establish a prediction model for irreversible II in SBO using objective factors (besides contrast-enhanced CT findings).

## Methods

### Patients

This retrospective study was approved by the Tokyo Medical University Hachioji Medical Center Ethics Committee (approval number TS2020-0358) and was conducted in accordance with the principles outlined in the 1964 Declaration of Helsinki and its later amendments. The need for informed consent was waived in view of the retrospective study design by the Tokyo Medical University Hachioji Medical Center Ethics Committee. We examined the data of 89 patients diagnosed with SBO by clinical symptoms and CT findings of closed-loop obstruction who underwent emergency operation between January 2014 and March 2022 at our department. Patients with large bowel obstruction, intraperitoneal malignancy, and a history of ascites were excluded. Finally, 83 patients were included, and all of these were confirmed for closed-loop obstruction due to mesenteric torsion or internal herniation at operation. Of these, 42 had irreversible II at laparotomy and required bowel resection (resection group; n = 42), whereas 41 had reversible II and required lysis of adhesions but not bowel resection (non-resection group; n = 41). We compared the clinical outcomes between the resection and non-resection groups.

### Perioperative clinical variables

Preoperative clinical variables included sex, age, body mass index, previous history of laparotomy, vital signs (systolic blood pressure < 100 mmHg, body temperature ≥ 38 °C), blood gas analysis (pH, base excess), and laboratory data, including white blood cell (WBC) count and C-reactive protein, hemoglobin, platelet, albumin, bilirubin, creatinine, and creatine kinase levels. Operative clinical variables included laparoscopic surgery, intraoperative presence of hemorrhagic peritoneal fluid, operation time, and blood loss. In addition, pathological findings of resection specimens included ischemia, mucosal hemorrhagic necrosis, and transluminal hemorrhagic necrosis.

### Radiographic variables

**Fig. 1:**
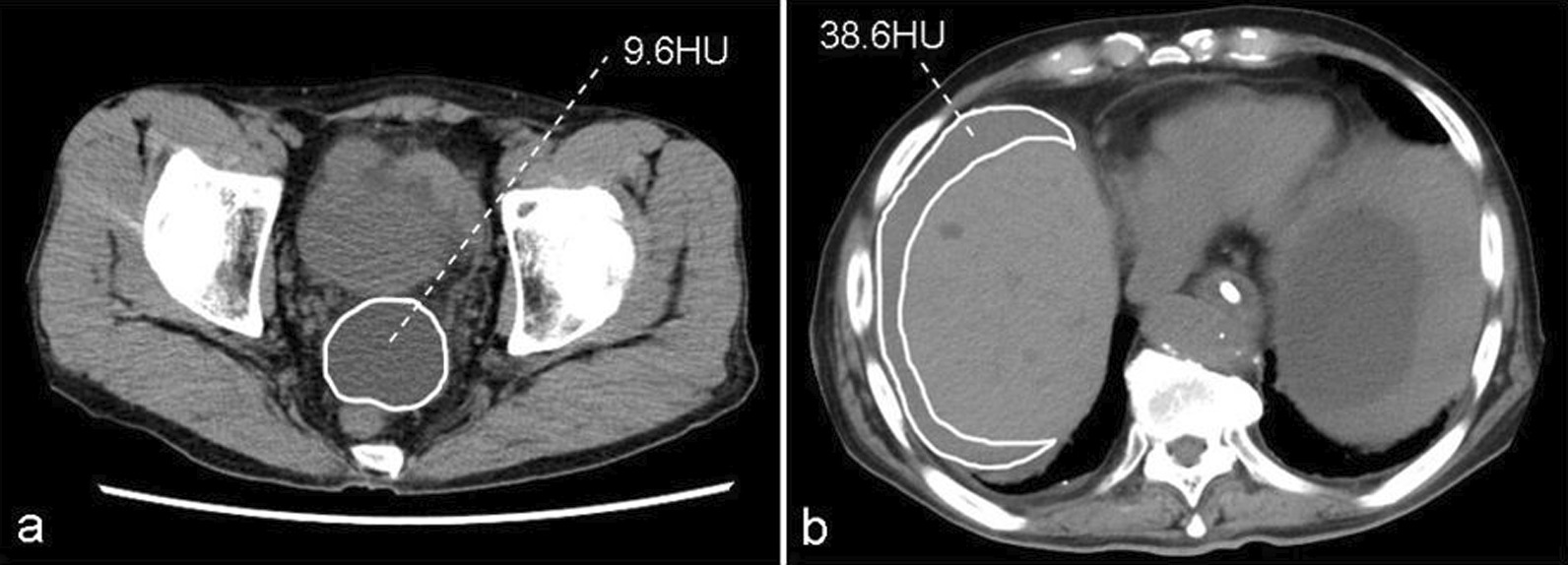
CT values of peritoneal fluid. Free peritoneal fluid in the rectovesical pouch (**a**) and below the right diaphragm (**b**) on non-enhanced computed tomography (CT) with mean CT values of 9.6 Hounsfield units (HU) and 38.6 HU, respectively, using a region-of-interest analysis

Radiographic variables included presence of free peritoneal fluid around the strangulated intestine, emphysema, the mean CT value of the free peritoneal fluid on unenhanced CT, and reduced bowel wall enhancement on contrast-enhanced CT. The mean CT value of the free peritoneal fluid was expressed in Hounsfield units (HU) and calculated as the mean of the region of interest by outlining the free peritoneal fluid without adjacent structures and gases at the maximum area of free peritoneal fluid on the axial section of the CT image (Fig. 1a, b).

### Statistical analysis

Statistical analyses were performed using the IBM SPSS Statistics, version 27.00 (IBM Corp., Armonk, NY, USA). In univariate analysis, the clinical and radiographic variables were compared between the resection and non-resection groups. The Mann–Whitney U test was used to examine the differences in continuous data. Fisher’s exact test or the χ^2^ test was used to compare categorical data between groups. *P* < 0.05 was considered statistically significant.

According to univariate analysis, preoperative clinical and radiographic variables (except for contrast-enhanced CT findings) with *P* < 0.05 were included in the multivariate analysis. Forward stepwise logistic regression analysis identified independent predictors of irreversible II necessitating bowel resection, and a final logistic regression model was obtained. Continuous variables were converted to binary variables at a cutoff value with the maximum Youden index, and a new prediction model was established. This model calculated the prediction score as the sum of the scores assigned to the binary variables from the independent predictors corresponding to the regression coefficient. To assess the discrimination and calibration of the prediction model, receiver operating characteristic (ROC) analysis and the Hosmer–Lemeshow test were used. To avoid overfitting, this model was evaluated using fivefold cross-validation. The rate of bowel resection, sensitivity, specificity, and positive and negative likelihood ratios were estimated for each score. Finally, using logistic regression analysis, odds ratios (ORs) and 95% confidence intervals (CIs) were evaluated between groups categorized by the best cutoff score from the ROC analysis. The power of the two-tailed independent t-test at 5% alpha was estimated to compare the model score for the groups by using a post hoc power analysis.

## Results

### Univariate and multivariate analyses of the resection and non-resection groups

The clinical and radiographic characteristics of the resection and non-resection groups are summarized in Table 1. There were no significant differences in patient characteristics or vital signs between groups. In blood gas analysis, base excess was lower in the resection group than in the non-resection group (− 1.7 mEq/L vs. 0.35 mEq/L; *P* = 0.006). Regarding laboratory data, only WBC counts were significantly higher in the resection group than in the non-resection group (12,550/µL vs. 9100/µL; *P* < 0.001).

**Table 1:** Comparison between the resection and non-resection groups

	Resection group (n = 42)	Non-resection group (n = 41)	*P*-value
Patient characteristics			
Age	75.5 (21–94)	72 (26–96)	0.433
Male sex	15 (35.7%)	22 (53.7%)	0.1
Body mass index (kg/m^2^)	19.9 (15.0–27.1)	20.4 (13.5–27.5)	0.909
Previous history of laparotomy	32 (76.2%)	31 (75.6%)	0.951
Vital signs			
Systolic blood pressure < 100 mmHg	4 (9.5%)	1 (2.4%)	0.187
Body temperature > 38 °C	3 (7.1%)	3 (7.3%)	0.651
Blood gas analysis			
pH	7.429 (7.219–7.575)	7.438 (7.23–7.542)	0.31
Base excess (mEq/L)	−1.7 (− 14.7 to 6.3)	0.35 (− 17.8 to 8.5)	0.006
Laboratory data			
WBC (/µL)	12,550 (4850–34,000)	9,100 (3060–20,600)	< 0.001
CRP (mg/dL)	0.73 (0.02–28.84)	0.27 (0.02–33.16)	0.36
Hb (g/dL)	13.2 (7.4–20.6)	13.8 (8.3–22.1)	0.689
Plt (×10^4^/µL)	25.7 (8.2–74.5)	22.6 (7.6–48.2)	0.515
Alb (g/dL)	3.5 (2.1–5.0)	3.7 (1.7–4.5)	0.45
Bil (mg/dL)	0.85 (0.2–1.7)	0.8 (0.3–7.0)	0.416
Cre (mg/dL)	0.735 (0.30–5.31)	0.75 (0.31–2.84)	0.743
CK (IU/L)	75 (20–1048)	81 (10–1156)	0.996
CT findings			
Presence of peritoneal fluid	42 (100%)	30 (73.2%)	< 0.001
Presence of emphysema	1 (2.4%)	0 (0%)	0.506
Poor or no contrast bowel wall enhancement^a^	22 (56.4%)	6 (17.1%)	< 0.001
CT value of peritoneal fluid (HU)	21.4 (10.2–77.0)	15.1 (6.7–32.5)	< 0.001
Operative factors			
Laparoscopic surgery	7 (16.7%)	15 (36.6%)	0.04
Presence of hemorrhagic peritoneal fluid	37 (88.1%)	14 (34.1%)	< 0.001
Operation time (min)	104 (53–291)	74 (36–143)	0.002
Blood loss (mL)	100 (10–2425)	10 (10–1570)	< 0.001
Pathological findings			
Ischemia	3 (7.1%)	–	
Mucosal hemorrhagic necrosis	9 (21.4%)	–	
Transluminal hemorrhagic necrosis	30 (71.4%)	–	

Categorical data are expressed as percentages, and continuous data are expressed as median (min–max)

*WBC* white blood cell, *CRP* C-reactive protein, *Hb* hemoglobin, *Plt* platelet, *Alb* albumin, *Bil* bilirubin, *Cre* creatinine, *CK* creatine kinase, *CT* computed tomography, *HU* Hounsfield unit

^a^Contrast-enhanced CT scan was not performed in three patients in the resection group and six patients in the non-resection group

Unenhanced CT scans were obtained in all 83 patients, while contrast-enhanced CT scans were obtained in 74 patients, as 6 patients with severe renal function impairment and 3 with a history of allergy to iodinated contrast agents were excluded. The presence of free peritoneal fluid was the most common CT finding, present in 100% of the resection group and 73.2% of the non-resection group (*P* < 0.001). Reduced bowel wall enhancement was found in 56.4% of the resection group and 17.1% of the non-resection group (*P* < 0.001). The free peritoneal fluid CT value on unenhanced CT scans was significantly higher in the resection group than in the non-resection group (21.4 vs. 15.1 HU; *P* < 0.001).

Regarding operative variables, laparoscopic surgery was performed in 16.7% and 36.6% in the resection and non-resection groups, respectively, (*P* = 0.04), and hemorrhagic peritoneal fluid at operation was found in 88.l% and 34.1% of patients in the resection and non-resection groups, respectively (*P* < 0.001). Operation time was significantly longer in the resection group than in the non-resection group (104 vs. 74 min; *P* = 0.002), and blood loss was significantly higher in the resection group than in the non-resection group (100 vs. 10 mL; *P* < 0.001).

In addition, pathological analysis revealed findings of hemorrhagic necrosis in 92.8% of the resection specimens (transluminal hemorrhagic necrosis in 71.4% and mucosal hemorrhagic necrosis in 21.4%).

Base excess, WBC count, presence of free peritoneal fluid, and peritoneal fluid CT value on unenhanced CT scans, which are significant preoperative variables on univariate analysis, were included in multivariate analysis. The forward stepwise logistic regression analysis results showed that WBC count and the peritoneal fluid CT value on unenhanced CT were significant predictors of irreversible II, necessitating bowel resection. The areas under the ROC curve (AUCs) were 0.741 (95% CI 0.633–0.849) for WBC count and 0.750 (95% CI 0.639–0.862) for free peritoneal fluid CT value.

### Prediction model

The prediction model was based on the final logistic regression model. WBC count and free peritoneal fluid CT value were identified as independent predictors and were converted into binary variables at the cutoff values of 12,000/µL and 20 HU based on the maximum Youden’s index from the ROC curve, respectively. Patients without free peritoneal fluid were considered as having a free peritoneal fluid CT value of < 20 HU. The regression coefficients for the two binary variables in logistic regression analyses were 1.658 and 2.104, respectively. For convenience, the regression coefficient was rounded off to the nearest integer to generate scores for the two independent predictors; thus, 2 points were assigned to each variable (Table 2). The prediction score was calculated as the sum of the points for each of the two independent predictors and ranged from 0 to 4 (Table 3).

**Table 2:** Predictors of irreversible intestinal ischemia necessitating bowel resection

Variables	Regression coefficient	OR (95% CI)	*P*-value	Score
WBC (/µL) ≥ 12,000 (/µL)	1.658	5.249 (1.812–15.209)	0.002	2
CT value of free peritoneal fluid (HU) ≥ 20 (HU)	2.104	8.201 (2.633–25.537)	< 0.001	2

*WBC* white blood cell, *CT* computed tomography, *HU* Hounsfield unit, *OR* odds ratio, *CI* confidence interval

**Table 3:** Prediction model for irreversible intestinal ischemia necessitating bowel resection

Variables	Score	
	0	2
WBC (/µL)	< 12,000	≥ 12,000
CT value of free peritoneal fluid (HU)	< 20	≥ 20
The prediction score	0–4	

*WBC* white blood cell, *CT* computed tomography, *HU* Hounsfield unit

The AUC for the new prediction model was 0.814 (95% CI 0.720–0.908) and 0.807 (95% CI 0.622–0.993) after fivefold cross-validation, which indicated good discrimination. The Hosmer–Lemeshow test indicated adequate goodness of fit (*P* = 0.391). The post hoc power analysis showed a power of 100% based on 83 patients at a 5% alpha level.

The bowel resection rate and the model’s diagnostic performance are shown in Table 4. A score of 2 was set as the optimal cutoff score based on the ROC curve, and logistic regression analysis showed that a score of 2 or higher was strongly associated with irreversible II, necessitating bowel resection (OR = 15.938, 95% CI 5.086–49.95, *P* < 0.001).

**Table 4:** The rate of bowel resection and diagnostic performance of each score in the scoring model

Score	Number of patients	Rate of bowel resection (%)	Sensitivity (%)	Specificity (%)	LR+	LR−
0	33	15.2	100	0	1	–
2	30	66.7	88.1	68.3	2.78	0.17
4	20	85	40.5	92.7	5.53	0.64

LR+: positive likelihood ratio; LR−: negative likelihood

## Discussion

In this study, we established a prediction model for irreversible II necessitating bowel resection in cases of SBO. Our model is based on two independent objective predictors: the WBC count and the value of free peritoneal fluid on unenhanced CT.

Systemic inflammatory response syndrome is reportedly associated with SBO [6, 8–12]. Particularly, only high WBC counts have been associated with irreversible II, reflecting the severity of inflammation due to the irreversible ischemic changes found in SBO [10]. Consistent with previous studies, the WBC count was significantly higher in the resection than in the non-resection group in the present study, and the AUC was 0.741, indicating a relatively accurate prediction of irreversible II.

Various CT findings, such as reduced bowel wall enhancement, increased unenhanced bowel wall attenuation, and the presence of mesenteric fluid, have been associated with irreversible II in cases of SBO [5–7, 13–15]. In our study, the value of the free peritoneal fluid on unenhanced CT was a significant predictor of irreversible II. SBO is caused by venous occlusion due to compression of the mesentery, causing transmural hemorrhage following congestion, edema, and mucosal hemorrhage. Thus, hemorrhagic peritoneal fluid is often observed in SBO. Kobayashi et al. reported that the presence of red blood cells in the free peritoneal fluid in SBO increases according to the degree of strangulation, and the count was higher in patients with bowel resection than in those without [16]. In our study, hemorrhagic peritoneal fluid during surgery was observed more frequently in the resection than in the non-resection group, which may reflect the transmural hemorrhage due to strangulation. Previous studies have reported CT values of the exudative body fluids < 10 HU [17], while those of hemorrhagic peritoneal fluid range from 15 to 75 HU [18]. A recent study reported that CT values of peritoneal fluid > 10 HU in cases of SBO indicate the need for bowel resection [19]. It has been suggested that the greater the hemorrhage associated with the progression of II due to strangulation, the higher the CT values of free peritoneal fluid. In our study, the AUC was 0.750, indicating a relatively accurate prediction of irreversible II.

The two indicators in our prediction model are objective indicators, allowing for objective and reproducible prediction of irreversible II in SBO. Additionally, these indicators are readily available at most hospitals. Furthermore, the CT value of free peritoneal fluid can be quickly evaluated using only unenhanced CT scans. Reduced bowel wall enhancement on contrast-enhanced CT has been reported as being helpful in predicting irreversible II in SBO, with a sensitivity of 75–81% and specificity of 19–74% [5, 7, 14]. However, contrast-enhanced CT may be contraindicated in patients with severe renal dysfunction or iodine allergy. In contrast, our model is useful even when contrast agents are contraindicated.

In our model, higher scores were associated with a higher probability of bowel resection. Accordingly, we believe that this model might help surgeons in recognizing the severity of the situation and in selecting the appropriate surgical procedure. For example, at a score of 0 (with a resection probability of 15.2%), immediate surgery may avoid a bowel resection for many patients with SBO. Moreover, it may be sufficient to release the strangulation, allowing for the choice of laparoscopic surgery. In fact, in this study, laparoscopic surgery was performed significantly more often in the non-resection group than in the resection group. At a score of 2 (with a resection probability of 66.7%), laparoscopic surgery may be prioritized, keeping in mind that open surgery might be required due to resection of ischemic bowel and patient deterioration. Conversely, at a score of 4 (with a resection probability of 85%), open surgery may be preferable considering the need for emergency resection of the ischemic bowel immediately before patient deterioration. In addition, respiratory and circulatory management can be considered preoperatively as a precaution against further deterioration in patients with progressive intestinal ischemia. Furthermore, higher scores were also associated with higher rates of necrosis in the resection specimens. All 17 specimens with a score of 4 in the resection group showed pathological hemorrhagic necrosis, and of them, 14 (82.4%) specimens showed transluminal hemorrhagic necrosis (data not shown).

Our study has several limitations. This was a single-center retrospective study with a small sample size. Multi-center prospective studies with large sample sizes are required to support the findings. To reduce overfitting, fivefold cross-validation was used to assess the internal validation of our prediction model. However, additional external validation is necessary to verify its application.

## Conclusion

In conclusion, we established a prediction model for irreversible II in cases of SBO based on objective variables, namely, the WBC count and the CT value of free peritoneal fluid on unenhanced CT scans. Our model can be made readily available in most hospitals and may enable surgeons to recognize the severity of the situation, prepare for the deterioration of patients with progression of intestinal ischemia, and select the appropriate surgical procedure for treatment.

## Data Availability

All data generated or analyzed during this study are included in this article. Further inquiries can be directed to the corresponding author.

## Abbreviations

AUC: Area under the receiver operating characteristic curve
CI: Confidence interval
CT: Computed tomography
HU: Hounsfield units
II: Intestinal ischemia
OR: Odds ratio
ROC: Receiver operating characteristic
SBO: Strangulated bowel obstruction
WBC: White blood cell
